# Valued Traits of Physician Leaders: A Comparative Study of First-Year and Final-Year Medical Students’ Perceptions

**DOI:** 10.1177/23821205251355072

**Published:** 2025-06-30

**Authors:** Sari Huikko-Tarvainen, Timo Tuovinen, Petri Kulmala

**Affiliations:** 1 60653 Faculty of Medicine, University of Oulu, Oulu, Finland; 2 667759 Research Unit of Health Sciences and Technology, University of Oulu, Oulu, Finland; 3 577217 Medical Research Center, Oulu University Hospital, Oulu, Finland

**Keywords:** Medical education, medical student, physician, physician leadership, traits

## Abstract

**Purpose:**

Leadership education in medical curricula lacks clear guidelines on which specific leadership skills should be emphasized at different stages. This study examines the valued traits of physician leaders as perceived by first- and final-year medical students, aiming to support stage-specific leadership education.

**Methods:**

In 2021, online questionnaires were administered to first- and final-year medical students, with participation rates of 90% (104/116) and 79% (86/109), respectively. Responses to the open-ended question, “How would you describe a good physician leader?” were analyzed using qualitative inductive content analysis to identify valued traits, followed by thematization and comparative analysis between groups. Furthermore, trait frequency was quantified to assess its prevalence.

**Results:**

Both groups shared core values, but the emphasis of specific traits shifted as students progressed through medical school. While both groups valued communication, fairness, approachability, empathy, and professionalism, final-year students placed greater emphasis on decisiveness, authority, and the ability to navigate complex clinical and administrative challenges. This comparison underscores the evolving understanding of leadership throughout medical education.

**Conclusions:**

These findings suggest that leadership education in medical curricula would benefit from a gradual and adaptive approach that aligns with students’ evolving needs. Early-stage medical education should emphasize interpersonal skills, communication, emotional intelligence, accountability, and ethical decision-making. As students gain clinical experience, the focus should broaden to include decisiveness, strategic thinking, operational management, and decision-making under pressure. Integrating leadership training progressively throughout medical education, with increasing exposure to ethical and practical leadership challenges, may better prepare students for the complexities of contemporary healthcare leadership roles.

## Introduction

Leadership is essential to organizational success, particularly in healthcare, where physicians are responsible for guiding teams and ensuring patient well-being and safety.^1^ Furthermore, in healthcare, formal leaders play a vital role in fostering employee engagement across different leadership functions.^2^ A systematic review of healthcare leadership has identified three core competencies: healthcare context-related, operational, and general competencies, with knowledge being the most frequently noted, alongside skills and attitudes.^3^ However, technical competence alone does not suffice; effective leaders must also inspire trust, foster teamwork, and support organizational success.^1,4,5^

Effective leadership involves diverse traits, including attributes and behaviors that reflect virtues and classical values such as trust, compassion, courage, justice, wisdom, temperance, and hope,^5^ along with empathy, integrity, and humility.^1^ Emotional intelligence—the capacity to manage one's own emotions while understanding others’—is considered essential for physician leaders^6,7^ and is often cited as the most important value for healthcare leadership.^1,5^ Emotional intelligence fosters trust among healthcare stakeholders,^8^ and empathy, a fundamental leadership value, enhances leaders’ ability to understand others’ perspectives, thereby improving communication.^1,4^ Decisiveness—often tied to courage and responsibility—is also vital, enabling leaders to manage teams effectively and ensure timely clinical decisions.^3,5^ Together with values like fairness and professionalism, decisive leadership enhances both team performance and patient care.^5^

Virtues constitute a foundational element of moral philosophy and provide a valuable framework for understanding leadership development.^9^ Central to virtues is their role in fostering self-knowledge and self-control^9^ Unlike values, which reflect the principles individuals consider important, virtues are qualities honed through consistent practice and conscious choice rather than mindless habit, guiding individuals toward morally correct actions.^9^ As such, virtues are typically understood as (a) dispositional, (b) deeply ingrained, (c) habitual qualities that (d) contribute to personal flourishing and (e) yield actions that are (f) performed well, (g) not poorly executed, and (h) motivated by the right reasons.^10^

### Leadership Education in Medical Curricula

Leadership competence is widely recognized as fundamental in medical education and healthcare settings.^11^ While there is consensus that leadership education should begin in undergraduate medical education,^12^ and recommendation advocate for leadership development that evolves in parallel with students’ clinical experiences,^13–15^ only a minority of medical schools worldwide formally include it.^16,17^ In countries like Finland, leadership studies are mandatory in medical curricula^18^; however, there are no clear guidelines on which skills to teach at each educational stage.^19^ Furthermore, previous research has identified training gaps in residents’ leadership education^20^ and confirmed the need for structured leadership development in academic medicine.^21^ Understanding perceptions of leadership across different levels of medical education can help shape curricula to meet students’ evolving needs. This study aims to inform leadership education structures by examining the traits valued by first-year and final-year medical students.

### Theoretical Framework

To facilitate the interpretation of and discussion on the results in the context of healthcare, this study employs three leadership theories as its theoretical framework: transformational leadership, ethical leadership, and servant leadership. Each theory offers a unique perspective on the role of traits in effective leadership, providing distinct views on what constitutes good leadership.^9^ Their inclusion is guided by their relevance to physician leadership.

### Transformational Leadership Theory

Transformational leaders empower subordinates by enhancing their sense of efficacy and purpose, encouraging collective work toward common goals.^22^ Such leaders inspire “trust, admiration, loyalty, and respect” in followers by appealing to their moral values and elevating ethical awareness.^22,23^ Through confident and optimistic behavior, transformational leaders communicate a vision for the organization and demonstrate its feasibility, thereby positively influencing subordinates’ experiences and outcomes.^24^

### Ethical and Servant Leadership Theory

Ethical and servant leadership both emphasize external guidance alongside internal commitment.^25^ Ethical leaders, perceived as truthful, caring, and principled, establish and model ethical standards^26^ that foster mutual trust and respect, resolve conflicts, and promote adaptive, welfare-enhancing problem-solving.^23^ Similarly, servant leaders prioritize followers’ development and well-being, advocating for what is ethically right beyond the organization's financial interests.^23^ This approach is especially relevant in healthcare, where leaders frequently navigate moral dilemmas such as resource allocation and balancing patient care with financial constraints.^3,5^ Leaders are responsible for mentoring, coaching, and training subordinates, modeling the values they endorse, and fostering critical thinking to identify effective solutions. These theories propose that leaders develop a vision incorporating subordinates’ input, values, and needs, and demonstrate a willingness to take personal risks and make decisive decisions to realize that vision.^23^

### Research Gap to Fill

To the authors’ knowledge, no previous study has explored whether medical students’ perceptions of valued traits evolve throughout their education. To address this research gap, it is essential to explore medical students’ perceptions of leadership by comparing first-year and final-year medical students’ views. People's behavior, actions, and motivation can be influenced by their perceptions.^27^ Furthermore, by identifying valued leadership traits and understanding medical students’ expectations of leaders, leadership education in medical curricula can be better tailored to prepare students for leadership challenges, enhance their followership, and support the development of leadership education that fosters these qualities in future healthcare leaders. Additionally, these findings can provide broader insights to inform stage-specific leadership training in medical curricula.

A notable aspect of the Finnish healthcare system is the opportunity for medical students who have completed their first four years of study to temporarily work in formal doctor vacancies under specific circumstances while pursuing their medical studies.^28^ This practice is legitimized by Finnish law^29^ and a very common aspect of Finnish medical education,^28^ in which medical students participate with very high frequencies.^30^ Given that final-year medical students in Finland have worked as doctors, whereas first-year students have not,^29^ the final-year medical students have also been led by physician leaders, but the first-year students have not. Thus, Finland provides a unique context to investigate how perceptions regarding physician leadership evolve during medical studies.

## Methods

### Study Design

In 2021, internet-based questionnaires were administered to first- and final-year medical students, achieving participation rates of 90% (104/116) for first-year and 79% (86/109) for final-year (sixth year) students. The full survey comprised multiple questions exploring students’ perspectives on physician leadership, various aspects of doctors’ work experiences (specifically their paid employment as doctors) during medical education, as well as demographic information, including respondents’ age, gender, and prior educational qualifications before entering medical school. However, these aspects were not included in the present study.

The open-ended question analyzed in this study was: “How would you describe a good physician leader?” This single, qualitative question was selected to allow for a broad range of responses, unrestricted by specific or narrowly defined issues. The responses were digitally saved and systematically coded to facilitate analysis. In total, the answers amounted to eleven A4-sized pages (Calibri font, 12-point, single-spaced).

A mixed-methods approach was employed to explore and answer the research question from multiple perspectives.^31^ First, responses were analyzed using qualitative, inductive content analysis to identify valued traits, followed by thematization, and the results were then compared between groups. Furthermore, the frequency of traits was quantified to assess their occurrence rates within each group.

To ensure reliability and validity, various forms of triangulation were utilized. These encompassed a mixed-method approach to validate the findings, the application of multiple theoretical perspectives to explain, interpret, and understand the results, and collaboration among multiple researchers who analyzed the empirical data and cross-verified their interpretations and conclusions.^32^ The authors, who are experts in medicine, medical education, and physician leadership research, contributed substantial expertise from academic, clinical, and physician leadership contexts. Additionally, employing a mixed-method approach facilitated a comprehensive examination of the qualitative material, enabling both depth and breadth of analysis, thereby strengthening validity and reliability.^32^

Inductive content analysis and thematization were chosen as they allow for qualitative data to be examined through emergent patterns, themes, and categories without imposing preconceived frameworks.^33^ The analysis followed a three-phase process: first, the data was read repeatedly for familiarity, with key phrases and concepts highlighted and coded as they related to leadership traits. Next, these open codes were reviewed and consolidated into broader categories based on similarities. Finally, these categories were refined into higher-order themes representing the core traits identified by students, enabling comparisons between the two groups. Representative quotations were included to support interpretations, with first-year students cited as (S) and final-year students as (P).

Following the inductive content analysis, a quantification analysis was performed. In this process, the data was analyzed by calculating how many participants expressed the same view.^34^ For both student groups, the frequency of traits within each category was determined. That is, the absolute number (*n*) and percentage (%) of each trait category were calculated, enabling direct comparison of results between groups. Quantification analysis offers an additional perspective on interpreting qualitative data^35,36^ enables a more nuanced discussion,^33^ and enhances the triangulation and reliability of the study.^32^

Data triangulation was also strengthened by including both first- and final-year students, offering a comprehensive view of how leadership traits are perceived across different stages of medical education. Comparable findings reinforce the conclusions from both groups of the study, while differences allow for a deeper discussion regarding their meaning and context.^33^ These various methods were also used to explore and answer the research question from multiple perspectives.^31^ Given the relatively small sample size in each category, advanced statistical analyses were not conducted. Finally, the results were organized in tabular form for clarity.

### Ethical Statement

The study complies with national and international ethical standards for non-medical research involving human participants, adhering to the ethical principles set forth by the Finnish National Board on Research Integrity TENK (2019) and the European Union's data protection regulations.^37^ Ethical committee approval was not required under Finnish law and ethical guidelines; however, approval was obtained from the Faculty of Medicine according to institutional policies. A statement from the Ethics Committee of Human Sciences at the University of Oulu is included in Appendix. Data is available upon reasonable request. Participants received comprehensive instructions detailing the study's objectives, the voluntary nature of their involvement, confidentiality and anonymity assurances, and their right to withdraw or withhold data at any point. Informed consent for participation and publication was obtained from all participants prior to data collection, and data analysis was conducted without personal identifiers. No incentives were provided for participation.

## Results

From the responses of 104 first-year medical students (participation rate: 90%) and 86 final-year medical students (participation rate: 79%), several key traits were identified through inductive content analysis and thematic categorization. These traits, along with their definitions and representative quotations from student responses, are presented below. To quantify these traits, we calculated the frequency and percentage of each trait mentioned in student responses (see Tables 1 and 2 for a detailed breakdown).

**Table 1.** Summary of Traits Identified in First-Year Medical Students’ Responses, Presenting a Quantitative Breakdown of Frequency and Relevance Within This Group.

Trait	Description	Supporting Quote	Frequency (*n*)	Percentage (%)
Communication skills	Ability to clearly and effectively communicate with others.	*A good leader must have excellent communication and collaboration skills. (S112)*	32	30.8%
Professionalism	Demonstrating competence and dedication to ethical behavior.	*Professional, honest, trustworthy, and approachable. (S78)*	28	26.9%
Approachability	Being open and accessible for dialogue.	*Approachable and understanding of patients. (S19)*	26	25.0%
Respect for others	Valuing the opinions and work of others within the team.	*Respects subordinates. (S15)*	23	22.1%
Fairness	Treating all individuals equally, without bias.	*Fair, determined. (S57)*	21	20.2%
Collegiality	Working well in a team, treating subordinates as equals.	*Collegial, helpful, energetic, and empathetic. (S40)*	20	19.2%
Empathy and compassion	Displaying care and understanding toward patients and staff.	*Empathetic and understands the demands of the profession. (S75)*	19	18.3%
Decisiveness	Ability to make firm and confident decisions.	*Decisive but considers others’ viewpoints. (S83)*	18	17.3%
Trustworthiness	Being reliable and fostering trust within the team.	*Trustworthy and professional. (S96)*	15	14.4%
Humility	Recognizing one's own limitations and not placing oneself above others.	*Does not see themselves as above others. (S86)*	12	11.5%

*Source.* Authors’ own work.

**Table 2.** Summary of Traits Identified in Final-Year Medical Students’ Responses, Presenting a Quantitative Breakdown of Frequency and Relevance Within This Group.

Trait	Description	Example (Citation)	Occurrences (*n*)	Percentage (%)
Good listener	Actively listens to both colleagues and patients.	*A good listener, friendly, helpful, considers both the employee's and patient's interests. (P3)*	28	32.6%
Fairness	Treats colleagues equally and justifies decisions.	*Fair, can justify decisions, knowledgeable. (P20)*	22	25.6%
Approachability	Ensures team members feel comfortable approaching them with concerns or questions.	*Approachable, considerate, open to change. (P26)*	19	22.1%
Communication skills	Communicates clearly and maintains open dialogue with subordinates.	*Communicates clearly. (P55)*	13	15.1%
Empathy	Understands the emotional and professional needs of others.	*Empathetic, approachable, creative. (P28)*	11	12.8%
Knowledge and expertise	Possesses strong clinical knowledge and expertise, stays up-to-date, and can relate to the challenges of subordinates.	*Knows their field exceptionally well but is also willing to listen to new perspectives. (P31)*	10	11.6%
Supportiveness and collegiality	Actively supports the development, well-being, and growth of subordinates.	*Knows their physicians and aims to develop them.* (*P24)*	9	10.5%
Decisiveness with humility	Makes informed and timely decisions while considering multiple perspectives.	*Decisive, humane, systematic. (P7)*	7	8.1%
Transparency	Maintains transparency in leadership decisions and communicates expectations clearly.	*Maintains a culture of transparency. (P17)*	5	5.8%

*Source.* Authors’ own work.

The results indicate that both groups identified similar core traits for physician leaders, though the emphasis on specific traits evolved as students progressed through medical training. First-year students’ descriptions of leadership focused on interpersonal qualities and a more egalitarian approach, with less attention to the complexities of high-level decision-making and administrative responsibilities. While medical students continued to value core traits such as communication, fairness, approachability, empathy, and professionalism, final-year students placed greater emphasis on decisiveness, authority, and the ability to manage complex clinical and administrative challenges. Their descriptions reflect a more nuanced understanding of the challenges leaders face in clinical and administrative contexts, emphasizing the need for leaders who can balance decisiveness with compassion and authority with approachability. A comparative analysis of the findings between first-year and final-year medical students regarding the traits of a good physician leader is presented in Table 3.

**Table 3.** Comparison of the Results Between First-Year and Final-Year Medical Students on Traits of a Good Physician Leader.

Trait	First-year medical students (104/116)	Final-year medical students (86/109)
Communication skills	Emphasizing effective communication for teamwork and approachability. *A good leader must have excellent communication and collaboration skills. (S112)*	Remains essential, but with an emphasis on communication in complex decision-making and interdisciplinary coordination. *Communicates honestly about plans and what to expect. (P20).*
Fairness	Highlighting the importance of equality and just treatment. *Fair, determined. (S57)*	Fairness continues to be valued, with more focus on clinical decision-making and resource allocation. “*A leader must be fair in allocating resources and managing conflicts*. *Systematic and thorough, fairly distributes shifts and on-call duties without favoritism, ensures adequate staffing, and prevents consistent overtime due to understaffing. Ensures consultation opportunities for trainees and medical students*. *(P78).*
Approachability	Stressing the need for accessible and open leaders. *Approachable and understanding of patients. (S19)*	Approachability remains valued but is balanced with the need for authority and decisiveness. *Approachable, considers different needs, constructive. (P72).*
Empathy and compassion	Emphasizing leaders’ understanding of personal and professional challenges. *Empathetic and understands the demands of the profession. (S75)*	Empathy is tied to managing team morale and resilience in high-pressure situations. *Empathetic, able to see things from the perspective of the subordinate (and patient). A multi-skilled individual who manages the whole system well. (P88).*
Decisiveness	Appreciating leaders who make decisions but consult others first. *Decisive but considers others’ viewpoints. (S83)*	Emphasis on decisiveness in clinical and operational decision-making. *Can justify decisions, knowledgeable, dares to make decisions, takes feedback and learns from it, listens to what's said on the ground about practical work, and addresses issues. (P20).*
Humility	Seeing humility as essential, with leaders being open to feedback. *Does not see themselves as above others. (S86)*	Humility is appreciated, but final-year students also expect leaders to balance it with confidence. *A good physician leader is selfless, considers him/herself and others as equals, but is also courageous and* forward-thinking *(P106)*
Professionalism	Emphasizing ethical behavior, reliability, and competence. *Professional, honest, trustworthy, and approachable. (S78)*	Professionalism is linked to managing clinical and administrative responsibilities effectively. *In addition to a solid clinical background, a good physician leader also understands how to interact with people and lead them. (P29).*
Respect for others	Stressing the importance of treating all team members with respect. *Respects subordinates. (S15)*	Respect remains a core value, but it is tied to empowering teams and effective delegation. *Knows the strengths and weaknesses of subordinates, provides responsibilities and support accordingly… Takes care of their own and others’ well-being. (P31).*
Trustworthiness	Associate trustworthiness with reliability and integrity. *Trustworthy and professional. (S96)*	Trustworthiness is emphasized in terms of reliability in high-pressure situations and transparent decision-making. *Builds a culture of trust, is systematic. Trustworthy in every way, keeps promises. Always takes responsibility. Admits mistakes and learns from them. (P30).*
Collegiality	Emphasizing collaboration and equality within teams. *Collegial, helpful, energetic, and empathetic. (S40)*	Collegiality remains important, particularly in navigating complex team dynamics in healthcare settings. *Someone who is good at organizing, creative, collegial, with a good sense of humor. Needs to be firm but not overly serious or distant. (P73).*

*Source.* Authors’ own work.

## Discussion

### Shifts in Emphasis on Leadership Traits

This study explored perceptions of leadership traits among first-year and final-year medical students. The findings suggest that foundational leadership traits, such as communication, fairness, approachability, empathy, and professionalism, are valued from the early stages of medical education and remain essential throughout. However, as final-year students gain clinical experience, they tend to prioritize traits like decisiveness, authority, and a more nuanced view of professionalism. This shift likely reflects their growing awareness of the complexities of medical leadership, including time-sensitive decision-making, interdisciplinary collaboration, and the balance between clinical and leadership responsibilities. These findings are consistent with prior studies identifying key physician competencies,^38,39^ and essential leadership skills, including empathy, initiative, emotional and organizational awareness, and communication as core traits.^1,4,15^ The observed progression from a focus on interpersonal traits among first-year students to a balanced perspective that includes ethical and managerial traits among final-year students aligns with the framework of transformational leadership. Transformational leaders, characterized by empathy, charisma, and vision, connect with followers emotionally and inspire them to act beyond self-interest for collective benefit.^22–26^ They motivate and inspire while embodying decisiveness and accountability, qualities particularly essential in high-pressure environments like healthcare. In the following sections, we discuss the findings of this study in greater detail, contextualizing them within the existing literature.

### Communication and Approachability as Foundational Traits

Both first-year and final-year students identified communication and empathy as core leadership traits, which aligns with previous research highlighting effective communication as essential to healthcare leadership.^1,4,5,40^ First-year students emphasized clear, effective communication with subordinates, colleagues, and healthcare teams, often underscoring collaboration and approachability as key leadership qualities. In contrast, final-year students placed greater weight on a leader's ability to navigate complex, high-stakes decision-making, indicating a shift toward strategic communication. This progression from a general focus on interpersonal skills to a more operational approach, integrating communication with decisiveness and authority, reflects a deeper understanding of leadership within clinical and administrative contexts. Both groups viewed effective leadership as the ability to manage team dynamics while fostering high levels of collaboration. This aligns with research showing that effective communication promotes teamwork and interdisciplinary coordination, both essential for optimal patient care.^1,40^

Approachability, along with a leader's willingness to listen and engage, was closely linked to communication and was highly valued by both groups. First-year students viewed an accessible, open leader as crucial to a positive work environment, whereas final-year students saw approachability as one component of a well-rounded leadership profile that includes decisiveness and authority. They described an approachable leader as someone who commands respect and makes sound decisions while remaining accessible. This perspective aligns with the principles of servant leadership, which emphasize follower development, well-being, and ethical integrity beyond organizational interests.^23^ Additionally, it suggests that effective leaders develop a vision that incorporates input from subordinates, respecting their ideas, values, and needs.^23^

### The Role of Empathy and Emotional Intelligence

Both groups identified empathy as a key leadership quality, emphasizing the importance of emotional intelligence in leadership, which supports previous research linking effective leadership to enhanced team performance, reduced burnout, and improved patient safety.^41^ First-year students highlighted the leader's role in understanding the personal and professional challenges of both subordinates and patients, while final-year students focused on empathy's role in decision-making, particularly concerning medical staff members’ work-life balance. Compassionate leadership was associated with resilience and the ability to uphold team morale in challenging clinical settings. These findings align with research on emotional intelligence, particularly empathy, as a vital component of leadership in fields with strong interpersonal demands, such as healthcare.^1,4,5^ The shift in emphasis suggests that clinical experience fosters a broader understanding of leadership, one that encompasses empathy alongside the capacity to make ethically sound, complex decisions. This is consistent with research highlighting the importance of ethical leadership—integrity and decisiveness in morally complex situations.^23,25,26^

A lack of empathy, communication, and respect in leadership can lead to a culture where physicians feel undervalued and overworked, impacting mental health.^42^ Quality of life is intrinsically linked to leadership quality.^43^ Poor leadership exacerbates physician burnout, which is linked to work overload, lack of control, insufficient recognition, lack of support, unresolved conflicts, and perceived injustice.^42,44^

The emphasis on compassion and approachability in early medical education reflects a foundational view of leadership as relational, with leaders seen as sources of emotional support. This view aligns with servant leadership principles, emphasizing leaders’ responsibility to nurture followers’ growth and well-being through guidance and internal commitment.^25^ Servant leaders prioritize their followers’ development, uphold ethical standards,^23^ and bear responsibility for mentoring, coaching, and training, embodying the values they wish to instill.^23^

### Evolving Views on Collegiality and Humility

First-year students associated collegiality and humility with respect for others, particularly in treating subordinates fairly and recognizing the contributions of all team members. They viewed leadership as a cooperative role focused on promoting team equality, with leaders working alongside rather than above their subordinates and remaining open to feedback. For final-year students, collegiality remained essential for team harmony, with respect forming the foundation for trust and collaboration in clinical leadership.

Humility, linked to ethical leadership principles,^25,26^ was highly valued by both groups. Among final-year students, the emphasis shifted towards balancing humility with confidence, expecting leaders to recognize their limitations while asserting authority in complex clinical situations. This balance reflects their understanding of the need for leaders who inspire both trust and respect, aligning with transformational leadership theory.^22,23^

In clinical settings, leaders must combine humility and openness to feedback with assertiveness in decision-making to maintain team cohesion and ensure patient safety, which aligns with previous research.^1,4^ This perspective on transformational leadership encourages collective progress toward shared goals,^22^ through the confident and optimistic communication of the organization's vision and its path to achievement. Such leadership has also been shown to enhance team experiences and outcomes.^24^ Additionally, ethical and servant leadership qualities are evident in the focus on external guidance, internal commitment,^25^ and fostering mutual trust and respect.^23^

### The Ethical Dimensions of Leadership: Integrity and Accountability

In Finland, the first two years of medical school emphasize theoretical instruction, while medical students begin receiving increasing clinical exposure from the third year of medical school onward.^45^ Based on the results of this study, for first-year students, trustworthiness is closely tied to reliability and integrity in decision-making, with expectations that leaders should be dependable and honest. Final-year students also highly value trustworthiness but broaden it to include consistent performance under pressure and responsible, transparent risk management in clinical settings. This shift reflects their deeper understanding of leadership's ethical dimensions, where leaders model fairness, honesty, and transparency, fostering a culture of ethical behavior.^25,26^ With clinical experience, students encounter more ethical dilemmas and greater responsibilities, likely intensifying their appreciation for these traits. The importance placed on integrity aligns with servant leadership principles, which emphasize that leaders uphold values and moral clarity, even in complex situations.^23^ In Finland, final-year students are temporarily permitted to work in formal doctor vacancies under certain conditions while pursuing their studies,^28^ and as they transition into the professional workforce, integrity becomes essential for navigating clinical and ethical complexities and maintaining trust with teams and patients.

### Professionalism and Fairness as Core Leadership Qualities

Professionalism was identified as a core value by both first-year and final-year students. First-year students associated it with ethical conduct, competence, and reliability, viewing leaders as role models. In contrast, final-year students emphasized a more nuanced understanding of professionalism, balancing clinical skills with administrative responsibilities. They regarded professionalism as fundamental to managing the complexities of healthcare leadership, including systemic pressures and ethical challenges. These insights reflect existing literature, which highlights that professionalism is key to fostering trust and accountability in healthcare leadership.^4^

Fairness was also highly valued, with first-year students underscoring equality and justice, expecting leaders to avoid favoritism and treat all team members equitably. For final-year students, fairness in decision-making was central, aligning with previous literature, which argues that fairness in leadership—treating team members equally and ensuring equitable decision-making—holds particular importance in healthcare's hierarchical structure, where power dynamics require careful management to avoid conflicts.^4^ These traits embody ethical leadership, in which leaders are truthful, caring, and principled in their decisions, setting ethical standards and modeling behavior that reflects those standards.^26^ Ethical leaders foster mutual trust and respect, resolve conflicts, and promote adaptive problem-solving that enhances long-term welfare.^23^

### Decisiveness and Leadership in High-Stakes Environments

As medical students advance, their understanding of leadership expands to include decisiveness and accountability, consistent with studies on essential leadership competencies.^5,40,46^ In high-stakes environments like healthcare, leaders must project confidence to earn their team's trust and ensure adherence to decisions.^5^ This shift likely stems from students’ increasing exposure to real-world clinical challenges, where patient outcomes often depend on timely decisions and effective management of high-pressure situations. First-year students associated decisiveness with a leader's willingness to consider others’ viewpoints before acting, favoring a thoughtful, consultative approach. In contrast, final-year students linked decisiveness to bravery, viewing it as essential for immediate, authoritative leadership in urgent circumstances.

This appreciation for decisiveness aligns with research on healthcare-specific leadership skills, including strategic thinking, problem-solving, emotional intelligence, and the ability to manage complex clinical dynamics.^5^ In clinical contexts, delays in decision-making can have serious consequences, making courage and responsibility paramount.

The emphasis on decisiveness and authority is consistent with research on clinical leadership, which identifies it as vital for leading teams and ensuring patient safety.^3^ This also aligns with broader research emphasizing leadership's importance in healthcare environments, which frequently demand quick, effective decisions, especially in high-stakes situations.^3^ Exposure to healthcare's hierarchical structure likely sharpens final-year students’ appreciation for clear, decisive leadership as essential for clinical efficiency. Their views reflect ethical and servant leadership principles,^22,23,25,26^ and align with transformational leadership theory, which suggests that effective leaders inspire their teams while demonstrating courage in challenging situations.^22–24^ Recognizing decisiveness as a key leadership quality indicates that medical students are attuned to the practical realities of healthcare leadership, where balancing empathy with swift action is fundamental for success.^1^

### Strengths, Limitations, and Future Research

A key strength of this study is its comprehensive approach, utilizing data from 104 of 116 first-year medical students (a 90% participation rate) and 86 of 109 final-year medical students (79% participation rate), which are considered exceptionally high response rates.^47^ These robust sample sizes enhance the reliability of the findings and facilitate a nuanced comparison of leadership perceptions at different stages of medical education. The use of inductive content analysis allowed themes to emerge directly from participant responses, yielding rich qualitative insights into the traits attributed to effective physician leaders. Furthermore, we provided a detailed, clear, and easily replicable methodology for quantifying qualitative information from the responses.

However, several limitations must be acknowledged. Data were collected from students at a single medical institution in Finland, which may restrict the generalizability of the findings to other educational settings or geographic regions. Additionally, reliance on self-reported data may introduce bias, as participants might provide responses that reflect socially desirable traits rather than their genuine views. The conversion of qualitative information into frequency counts can decrease the depth of interpretation regarding participants’ experiences as communicated through the interviews.^48^ The study also did not consider demographic variables that may influence students’ perceptions. Interpreting the evolution or maturation of leadership perceptions over time is challenging when data are collected at a single point in time from two different cohorts. While the study offers valuable insights, the cross-sectional design does not allow for tracking changes within the same cohort over time. Therefore, future research could investigate the evolving perceptions of the same cohorts in a longitudinal study as they progress through their medical careers, providing deeper insights. As final-year medical students in Finland temporarily work as doctors in formal doctor vacancies under certain circumstances,^29^ and do so with very high frequencies,^30^ they have also been led by physician leaders, which first-year students have not. These legal, educational, and cultural aspects can shape students’ experiences and, thus, their perceptions of leadership. These differences may also reflect external influences, such as political or social events.

### Practical Implications for Leadership Education in Medical Curricula Lessons for Practice

The findings carry significant implications for leadership education in medical curricula. First-year students’ emphasis on interpersonal traits suggests that early leadership training should focus on foundational skills like communication, emotional intelligence, and accountability, which are essential for building trust and understanding with patients and colleagues. As students’ progress, leadership education should gradually incorporate advanced competencies, such as decisiveness, strategic thinking, operational management, and decision-making under pressure, equipping students for the complexities of clinical leadership.

By integrating leadership education throughout the medical curriculum, while gradually introducing ethical and practical challenges, future physicians will be better prepared for the demands and complexities of healthcare. Moreover, understanding the evolving leadership expectations of medical students can enhance both leadership and followership skills, ultimately benefiting patient care, as effective leadership directly contributes to improved patient safety outcomes.^41^

## Conclusion

Comparing first-year and final-year medical students’ perceptions of leadership traits reveals a more mature understanding of leadership as students advance in their education. While both groups valued communication, fairness, approachability, empathy, and professionalism, final-year students emphasized decisiveness, authority, and the ability to navigate complex clinical and administrative challenges. These findings highlight the need for flexible leadership styles to address diverse situations. Our study extends leadership research by underscoring the potential benefits of a dynamic, evolving approach to leadership education within medical curricula, one that grows alongside students’ advancing clinical experience.

## Supplemental Material

sj-docx-1-mde-10.1177_23821205251355072 - Supplemental material for Valued Traits of Physician Leaders: A Comparative Study of First-Year and Final-Year Medical Students’ Perceptions Supplemental material, sj-docx-1-mde-10.1177_23821205251355072 for Valued Traits of Physician Leaders: A Comparative Study of First-Year and Final-Year Medical Students’ Perceptions by Sari Huikko-Tarvainen, Timo Tuovinen and Petri Kulmala in Journal of Medical Education and Curricular Development

sj-docx-2-mde-10.1177_23821205251355072 - Supplemental material for Valued Traits of Physician Leaders: A Comparative Study of First-Year and Final-Year Medical Students’ Perceptions Supplemental material, sj-docx-2-mde-10.1177_23821205251355072 for Valued Traits of Physician Leaders: A Comparative Study of First-Year and Final-Year Medical Students’ Perceptions by Sari Huikko-Tarvainen, Timo Tuovinen and Petri Kulmala in Journal of Medical Education and Curricular Development
