# Supplementary material for: Valued Traits of Physician Leaders: A Comparative Study of First-Year and Final-Year Medical Students’ Perceptions
Source: J Med Educ Curric Dev. 2025 Jun 30;12:23821205251355072. doi: 10.1177/23821205251355072 (PMC12217562; doi:10.1177/23821205251355072)

Appendix. The statement from the Ethics Committee of Human Sciences at the University of Oulu.

The contact person for the Ethics Committee of Human Sciences at the University of Oulu is Janne Kurtakkko; e-mail: janne.kurtakko@oulu.fi


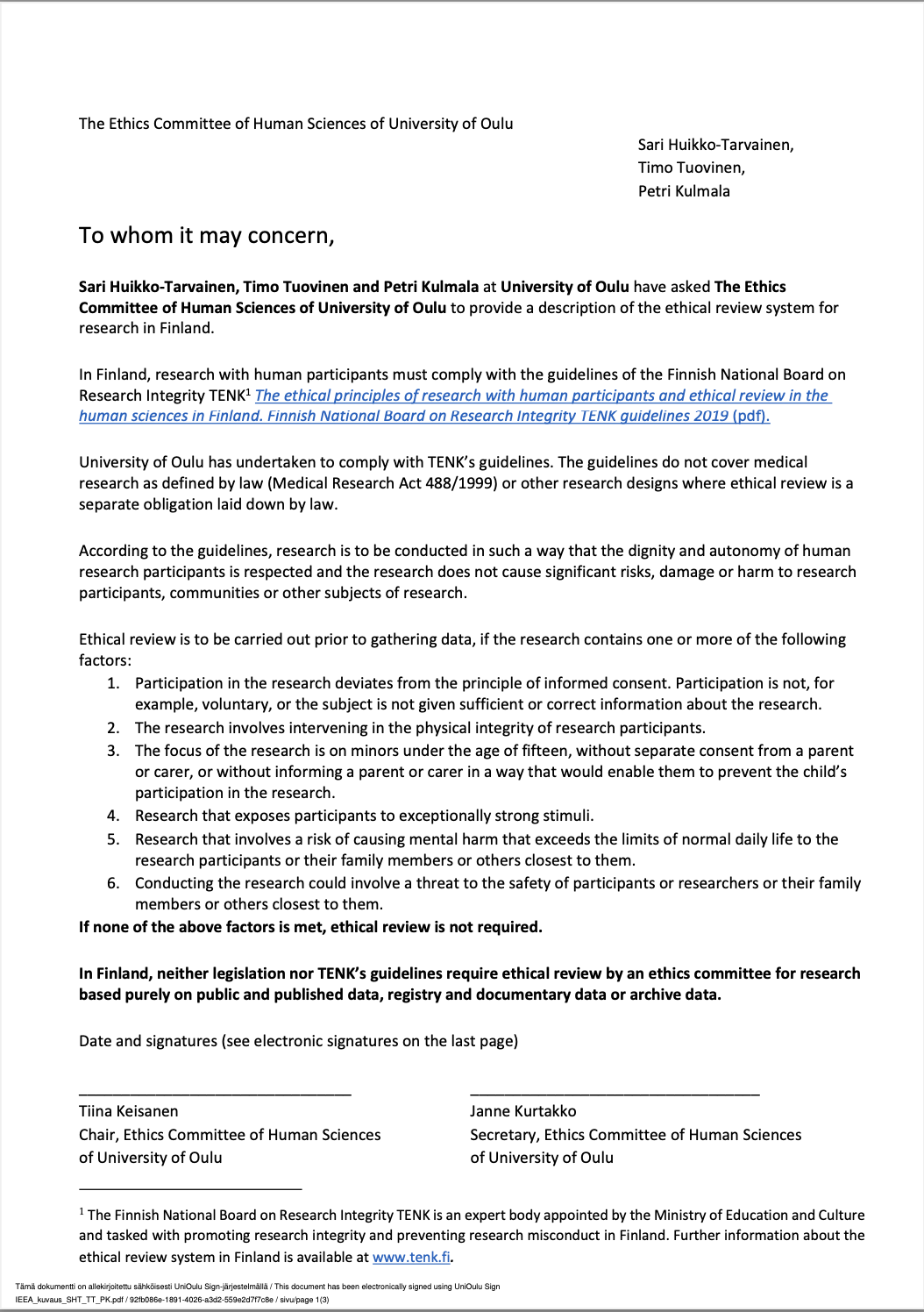


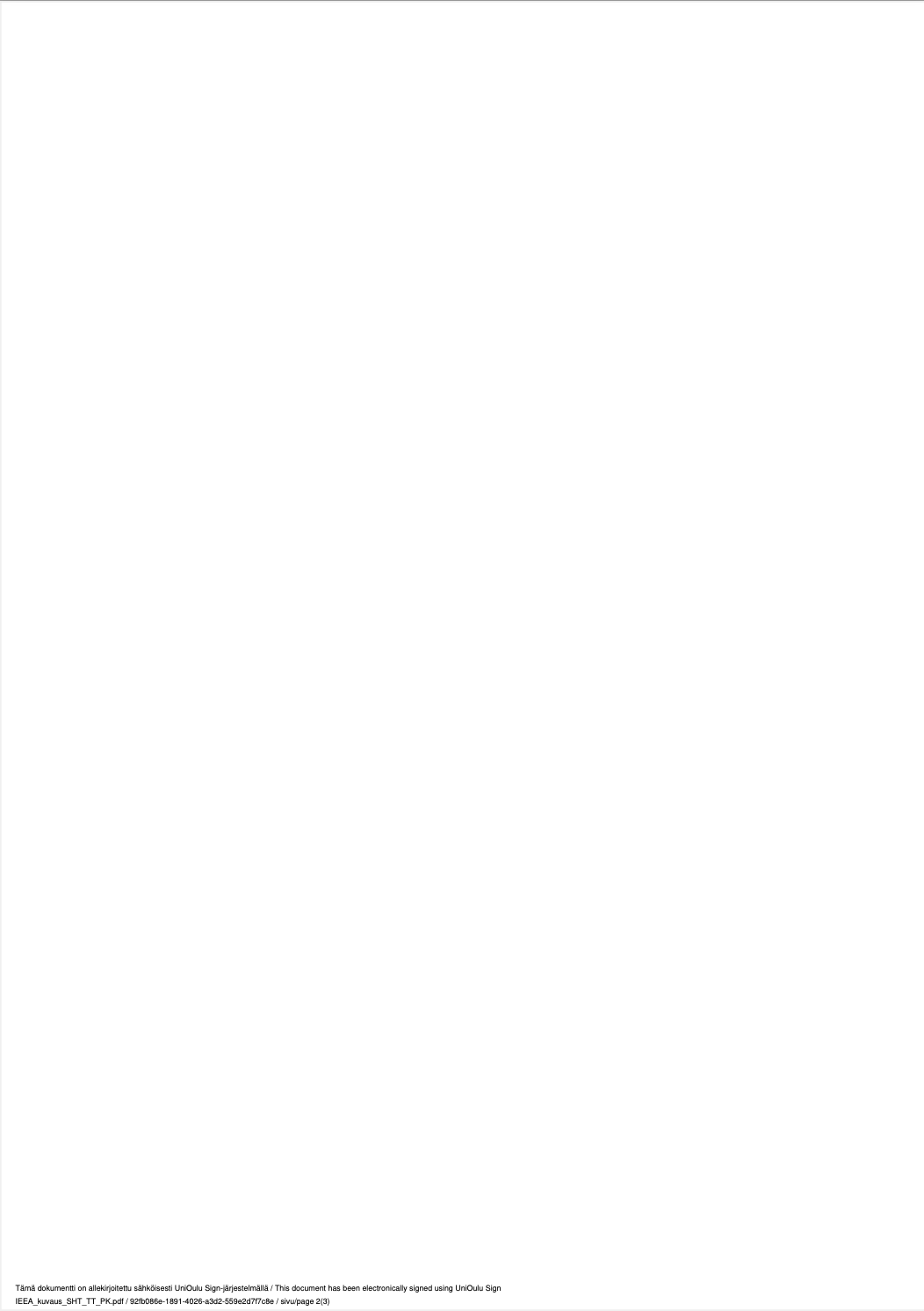


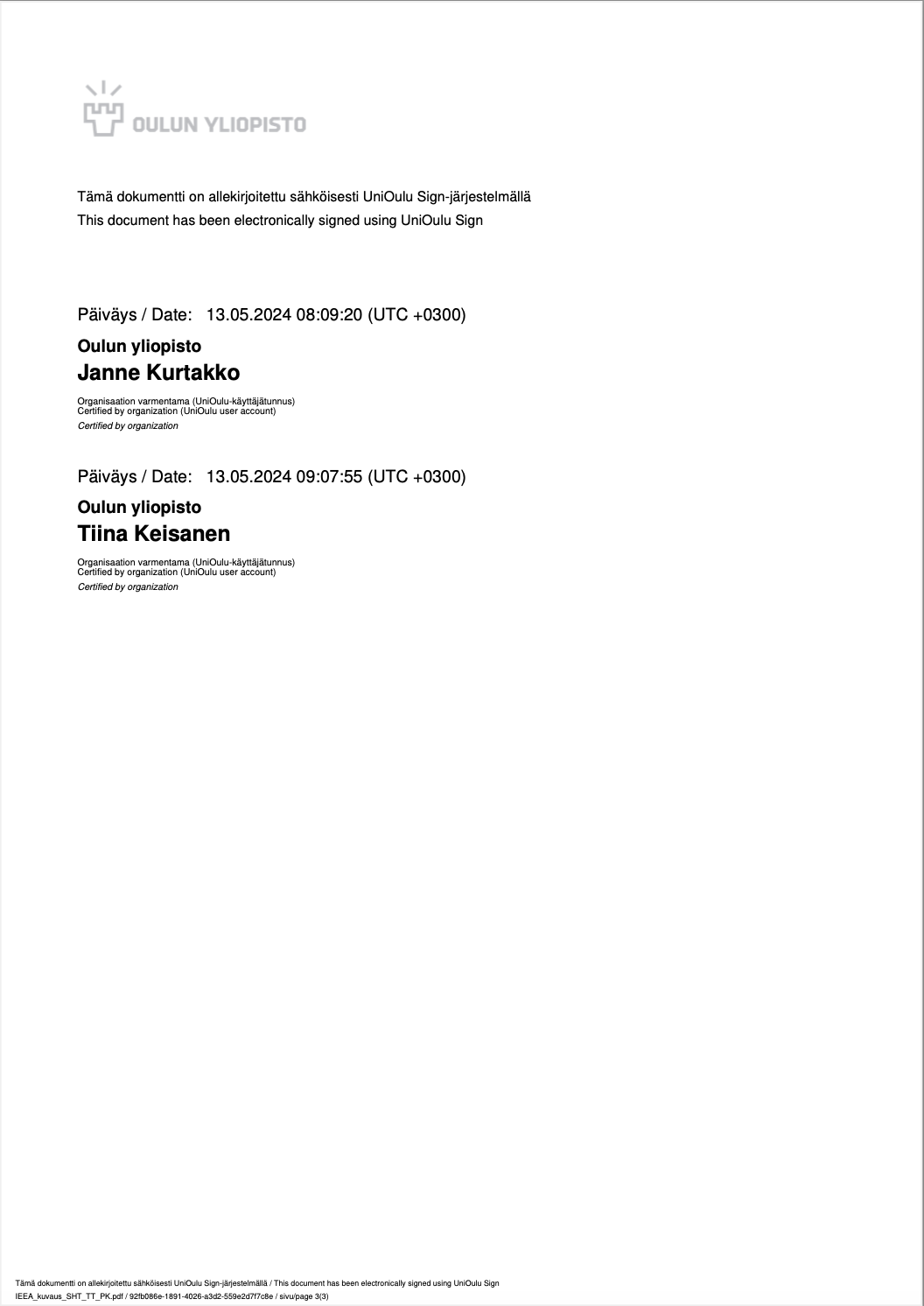

Supplement: sj-docx-1-mde-10.1177_23821205251355072 - Supplemental material for Valued Traits of Physician Leaders: A Comparative Study of First-Year and Final-Year Medical Students’ Perceptions [file sj-docx-1-mde-10.1177_23821205251355072.docx]
